# Cardiovascular disease: addressing poverty is key

**DOI:** 10.1016/j.lana.2025.101029

**Published:** 2025-02-14

**Authors:** The Lancet Regional Health – Americas

Cardiovascular disease is a group of clinical conditions that include ischemic heart disease (the most common cardiovascular disease), stroke, peripheral arterial disease, heart failure, arrythmias, and other less common cardiovascular disorders such as cardiomyopathies. Every year, nearly 18 million people worldwide die due to cardiovascular disease. As the global leading cause of morbidity and mortality, cardiovascular disease continues to be a high burden for the health-care system and the global economy, and remains a high priority for research funding institutions, investors on innovative technology and drug therapies, and the public health sector.

Cardiovascular mortality rates differ across regions. For example, data from 2022 show that the age-standardised mortality rate of ischemic heart disease was highest in central Asia (269 per 100,000) and eastern Europe (254 per 100,000) and lowest in high-income Asia Pacific (26 per 100,000). In the Americas, the Caribbean (105 per 100,000) and central Latin America (109 per 100,000) had the highest mortality rate compared with the rest of the Americas (59–76 per 100,000). In the USA, cardiovascular disease represents more than US$400 billion annually in direct and indirect costs. In Latin America, data are more scarce. An analysis in nine countries revealed that the economic impact attributed to heart attack, heart failure, atrial fibrillation, and hypertension combined was US$31 billion in 2015, which represents a considerable burden in countries with smaller economies. Given the high impact of cardiovascular disease on human lives and on nation economies, it is imperative to strengthen prevention measures.

In this issue of *The Lancet Regional Health — Americas*, we publish a Series on Cardiovascular Disease in the Americas. With two comprehensive Reviews, the Series covers epidemiology and prevention of cardiovascular disease in the region. The first paper, by Joseph and colleagues, provides an overview of the most current data available on the prevalence and incidence of cardiovascular disease and cardiovascular mortality rates in the region. It also covers the epidemiology of risk factors for cardiovascular disease. In the second Review, Schwalm and colleagues highlight innovative approaches to foster prevention and discusses barriers that are unique to many low-income and middle-income countries across the Americas.

Socioeconomic determinants of health can further undermine the efforts to address cardiovascular disease in nations with less affluent economies. For example, high-income Asia Pacific and central Latin America have similar age-standardised prevalence of ischemic heart disease (about 2600 cases per 100,000), but the mortality rate due to ischemic heart disease in central Latin America is four times that in high-income Asia Pacific (109 *vs* 26 per 100,000, respectively). These findings suggest that poverty might represent a major determinant of cardiovascular disease. For example, multidimensional poverty—inclusive of income, education, insurance status, and self-reported health—is strongly associated with atherosclerotic cardiovascular disease.

Poverty affects millions of people globally, even in high-income countries. In the USA, the poverty rate in 2022 was 11.5% (37.9 million people). According to the latest data available, the proportion of people living below the national poverty line is much higher for other countries in the region (for example, 59.3% in Guatemala [9.4 million people], 58.5% in Haiti [6 million people], 39% in Bolivia [4.6 million people], 32% in Argentina [8.9 million people], and 25.9% in Peru [8.6 million people]). Poverty can strongly influence householders to buy cheaper and less healthy food. Often, self-employed people must work longer hours to pay daily living expenses, having less time for physical activity. People with lower incomes live in neighbourhoods that usually have less infrastructure designed to promote physical activity (eg, sidewalks, bike lanes, community parks, community outdoor gyms, and outdoor lights for safe physical activity at night). Low income is also associated with less access to higher education. School absenteeism is also higher among families with very low income, as children join the workforce to support household income to afford living costs. This is a concern, as education is essential to understand the importance of disease prevention and awareness of the consequences of having cardiovascular disease and related risk factors (eg, smoking). Job insecurity can also contribute to perpetuating the aforementioned problems. People with cardiovascular disease who face economic hardship may even prioritise daily income over disease treatment. All this is in addition to known barriers related to inequity in health-care access and treatment (eg, lack of health insurance).

Commendable efforts and advances have been made to address cardiovascular disease and preventable risk factors (eg, diabetes, obesity, unhealthy diet, low physical activity, cholesterol, and hypertension), as noted in the papers by Joseph and colleagues and Schwalm and colleagues. In fact, most cardiovascular diseases are preventable (eg, ischemic heart disease and stroke), and adopting a healthy lifestyle is essential for reducing the risk of cardiovascular disease. However, many risk factors are undoubtedly determined by poverty and other socioeconomic and commercial determinants of health. Unless governments address poverty among their people and inequities in the health-care system, it is unlikely that we will make major and sustained improvements in reducing cardiovascular disease incidence and mortality rates.

To date, there is little information about the actual economic burden of cardiovascular disease for most countries in the Americas. It is also unclear the outcomes and feasibility of massive implementation of task shifting—proposed by the HEARTS in the Americas initiative to address cardiovascular disease at the primary care level—and the effectiveness of the use of digital health due to concerns about policy regulations, affordability, and digital literacy. *The Lancet Regional Health — Americas* invites original contributions to better understand the burden of cardiovascular disease in the region, the effectiveness of new proposed prevention policies, and the actual impact of poverty on cardiovascular disease.

